# Current Biologic Therapies for Severe Asthma and Real-World Data: Are Expectations Being Met?

**DOI:** 10.3390/jcm13237152

**Published:** 2024-11-26

**Authors:** Elena Villamañán, Daniel Laorden, Paula Granda, Carmen Sobrino, Susana De Andrés, Carlos Carpio, Javier Domínguez-Ortega, David Romero, Pablo Mariscal, Leticia De Las Vecillas, Santiago Quirce, Rodolfo Álvarez-Sala, on behalf of AsmaGrave-HULP Study Group

**Affiliations:** 1 Department of Pharmacy, Hospital La Paz, IdiPAZ, 28046 Madrid, Spain; grandap@gmail.com (P.G.); carmen.sobrino@salud.madrid.org (C.S.); deandres@gmail.com (S.D.A.); 2 Department of Pneumology, Hospital La Paz, Universidad Autónoma de Madrid, IdiPAZ, and CIBER of Respiratory Diseases, 28046 Madrid, Spain; laordendaniel@gmail.com (D.L.); carpio@gmail.com (C.C.); david.romero@salud.madrid.org (D.R.); pablo.mariscal@salud.madrid.org (P.M.); rodolfo.alvarezsala@salud.madrid.org (R.Á.-S.); 3 Department of Allergy, Hospital La Paz, IdiPAZ, and CIBER of Respiratory Diseases, 28046 Madrid, Spain; javier.dominguez@salud.madrid.org (J.D.-O.); leticia.vecillas@salud.madrid.org (L.D.L.V.); santiago.quirce@salud.madrid.org (S.Q.)

**Keywords:** biologic therapies, severe asthma, real world data

## Abstract

Advances in knowledge about clinical features, physiology, and underlying immunology are leading to targeted therapies and a new era of therapies. Biological treatments for severe asthma have changed the way this disease is managed, especially in patients who do not respond adequately to conventional treatments with corticosteroids and bronchodilators. These treatments block the action of different molecules involved in the immune response and in the inflammation of the airways, bronchoconstriction, bronchial hyperresponsiveness, and excessive mucus production. Currently, there are sufficient real-life data to corroborate the good results obtained in clinical trials by these type of drugs for severe asthma patients. Observational studies reveal their efficacy and safety, reducing exacerbations, leading to fewer emergency room visits and hospitalizations, and improving quality of life with better asthma control and better functional status.

## 1. Introduction

Asthma is an inflammatory disease that affects approximately 339 million people worldwide, causing 1000 deaths per day. Although the mortality rate has decreased by almost 59% in that same period, thanks to advances in treatments, some patients are unable to mitigate their symptoms and end up suffering from severe asthma (SA). The prevalence has increased from 1990 to 2015 by 12.6%. Affecting mainly middle-aged people and women, it can be explained by an increase in allergic asthma, with the stabilization of non-allergic asthma [1,2,3].

Studies such as the ISAAC (International Study on Asthma and Allergies in Chilhood), which surveyed a large number of children (1,200,000), with 98 countries participating, observed a prevalence of current asthma of 14.1% in the 13–14-year age group and 14.6% in the 6–7-year age group [4].

It is also a cause of a substantial burden of disease, reducing quality of life, in people of all ages in all parts of the world. Asthma continues to be a major source of global economic impact in terms of both direct and indirect costs. Avoidable asthma deaths are still occurring due to the inappropriate management of asthma, and this needs to be rectified [5].

Severe asthma is defined as asthma that is uncontrolled despite receiving treatment in the last year with a combination of high-dose inhaled glucocorticoids/long-acting β2 agonists (GCIs/LABAs) and long-acting anticholinergics (LAMAs) or which requires maintenance with oral corticosteroids (OCSs) (treatment lasting 6 months a year regardless of dose, or cumulative dose > 1 g of prednisone or equivalent, regardless of duration). The lack of control includes any of the following characteristics:

- Asthma Control Test (ACT) below 20 or Asthma Control Questionnaire (ACQ) cut point of 1.5 or greater.
- Two or more severe exacerbations or having received two or more courses of OCS (lasting three or more days each) in the previous year.
- At least one hospitalization for severe exacerbation in the previous year.
- Chronic limitation of airflow (forced expiratory volume ratio in the first second/forced vital capacity (FEV1/FVC) < 0.7 or FEV1 < 80% of predicted) after the use of appropriate treatment (as long as the best FEV1 is greater than 80%) [3].

These patients require closer monitoring than those with moderate or mild asthma do, and their health care is associated with a greater consumption of economic resources. Currently, severe asthma accounts for more than 50% of the attributable cost of asthma [4,5,6,7,8].

### 1.1. Relevant Section

Over the past 25 years, advances in knowledge about the clinical features, physiology, and underlying immunology of severe asthma have brought about a major shift in the understanding of the molecular mechanisms of the disease, leading to targeted therapies and a new era of biologics.

GINA [3] currently recognizes two asthma endotypes based on the degree of type 2 inflammation in the airways: type 2–high (T2-high) and type 2–low (T2-low) asthma. T2-high asthma is defined as the presence of one or more of the following signs in a patient taking high-dose inhaled corticosteroids:

- More than 150 per μLw blood eosinophils or more than 2% in sputum.
- FeNO over 20 ppb.
- Allergen-driven asthma.

The majority of asthma shows evidence of cytokines associated with T-helper 2 cell (T2)-mediated inflammation and is termed T2-high. The pathogenesis of T2-high asthma is chiefly orchestrated by interleukins (IL)-4, IL-5, and IL-13 and is usually accompanied by eosinophil infiltration. T2-high disease is clinically determined by elevated peripheral blood or sputum eosinophil levels using consensus-derived, non-type 2 inflammation (Non-T2)-mediated asthma, which is difficult to define due to a lack of signature biomarkers. It exists in the absence of T2-high or eosinophilic inflammation and includes neutrophilic and paucigranulocytic subtypes. Several cell types and cytokines, including Th1, Th17, IL-6, and IL-17, contribute to mechanisms of non-T2 asthma. Neutrophil extracellular traps (NETs) and inflammasome activation likely play a role in severe neutrophilic asthma. Several mechanisms lead to the uncoupling of airway hyperresponsiveness and remodeling from airway inflammation in paucigranulocytic asthma.

### 1.2. Current Biological Treatments in Severe Asthma and Real-World Data

Biological treatments for severe asthma have changed the way this disease is managed, especially in patients who do not respond adequately to conventional treatments with corticosteroids and bronchodilators. These treatments block the action of different molecules involved in the immune response and in the inflammation of the airways, bronchoconstriction, bronchial hyperresponsiveness, and excessive mucus production (Table 1).

#### 1.2.1. Anti-IgE Biologics

##### *Omalizumab* 

This is a humanized monoclonal antibody that selectively binds to human immunoglobulin E (IgE) and prevents its binding to the high-affinity IgE receptor on basophils and mast cells, thus reducing the amount of free IgE available to trigger the allergic cascade.

Its administration is subcutaneous and the administration schedule is determined from the basal IgE concentration (IU/mL), measured before starting treatment, and body weight (kg). Based on these determinations, 75 to 600 mg in 1 to 4 injections may be necessary [9].

Omalizumab does not have important interactions with other drugs, though, since IgE may be related to the immune response to some helminth infections. This drug may indirectly reduce the effectiveness of medications used to treat helminth infections or other parasites.

Since it was first marketed, almost 20 years ago, it has been shown to be a safe drug with mild adverse effects. The most frequent are headache and reactions at the injection site, which include pain, swelling, erythema, and pruritus [9].

Many real-world data (RWD) confirm the results released by randomized clinical trials (RCTs) [10,11,12,13,14,15]. Omalizumab has also proven in real life to improve the quality of life of patients with moderate/severe uncontrolled asthma and that this improvement is maintained over time [16]. Moreover, this drug can achieve a complete response in patients with allergic SA [17].

In a meta-analysis including 86 publications, Bourquet et al. [18] assessed clinical outcomes in the real world compared with pre-marketing randomized clinical trials (RCT) and corroborated all those findings. They observed consistent improvements in GETE, lung function, and PROs, as well as reductions in asthma exacerbations, OCS use, and HCRU with omalizumab in real life, which confirms and complements the efficacy data of RCTs.

#### 1.2.2. Anti-IL-5 and Anti-IL-5R Biologics

##### Anti IL-5 Biologics

*Mepolizumab*

This is a humanized monoclonal antibody that binds with high specificity and affinity to interleukin 5 (IL-5). This is the main cytokine that modulates eosinophils in blood and tissues. In this way, mepolizumab prevents the binding of IL-5 to the surface of eosinophils, which leads to a reduction in their production and survival. The regimen in adults is 100 mg administered subcutaneously, once every 4 weeks [19].

It is approved for patients with uncontrolled eosinophilic asthma with an Eos level of >500 or <500 with two severe exacerbations or one hospitalization in the previous year. Mepolizumab has shown more effectiveness in patients with more than 500 Eos/l, although efficacy has also been observed in patients with ≥300 Eos/l in the blood in the last year or with ≥150 Eos/l at the time of treatment but with a controlled history of eosinophilia [20,21].

Mepolizumab can be considered a safe drug with few relevant adverse effects; the most frequently reported adverse reactions are headache (20%), injection site reactions (8%), and back pain (6%). Moreover, it can be used concomitantly with other drugs, given its low potential for drug interactions [19].

Three clinical trials, DREAM151 [22], SIRIUS152 [23] and MENSA [24], showed that the drug reduced exacerbations and OCS use and improved quality of life.

Mepolizumab has shown a relative reduction in the risk of severe exacerbation of approximately 50% in patients who were inadequately controlled despite receiving high doses of ICS and/or OCS in the pivotal DREAM [22] and MENSA [24] studies. In absolute terms, the differences in annualized exacerbation rates with respect to the placebo were 1.16 exacerbations/patient/year in the first study and 0.91 exacerbations/patient/year in the second. Also, in the DREAM [22] and MENSA [24] studies, a greater reduction (73%) in the frequency of clinically relevant exacerbations were observed in the subgroups of patients with higher baseline plasma eosinophil levels (≥500/μL).

On the other hand, in the SIRIUS [23] study, 64% of patients treated with mepolizumab experienced some degree of reduction in their daily OCS dose, compared with 44% in the placebo group. These results were consistent across all subgroups, with the greatest reduction in the frequency of clinically relevant exacerbations being observed in those patients with higher baseline plasma eosinophil levels.

Since its commercialization, numerous real-life studies have evaluated its effectiveness and safety in patients with severe asthma, providing a broader perspective on its impact in clinical practice. As in clinical trials, RWD with this drug have demonstrated a reduction in exacerbations and improved lung function and quality of life assessed by specific questionnaires such as the Asthma Control Questionnaire (ACQ) and the Asthma Quality of Life Questionnaire (AQLQ). Furthermore, observational studies have also shown that mepolizumab allows for a significant reduction in the dose of oral corticosteroids necessary for asthma control, which translates into a more favorable side effect profile for patients [25,26]. The safety profile of mepolizumab in real-world settings is consistent with that observed in clinical trials [19,27,28].

*Reslizumab*

It is a humanized monoclonal antibody that specifically binds to IL-5, preventing its binding to the surface of eosinophils, which results in a reduction in their production and survival. It is administered intravenously at a dose of 3 mg/kg body weight [29].

No interaction studies have been performed with reslizumab but, considering its characteristics, no interactions are expected. The results of a population pharmacokinetic analysis confirm that the concomitant use of leukotriene antagonists or systemic corticosteroids does not affect their pharmacokinetics.

Its safety profile, like that of the previous ones, is good. Few adverse reactions have been reported, most commonly elevated blood creatine phosphokinase (approximately 2% of patients) and anaphylactic reaction [29].

The efficacy of reslizumab in eosinophilic asthma was studied in four pre-marketed randomized, double-blind, placebo-controlled phase III clinical trials—studies 3082 and 3083, 52 weeks in duration (pivotal studies), and studies 3081 and 3084, 16 weeks in duration (supporting studies)—as well as in an open-label, uncontrolled extension study (study 3085) [29]. Studies have shown that it is effective in patients with SA with an eosinophilic phenotype with ≥ 400 Eos/l. In a randomized placebo-controlled study, Castro et al. [30] observed that a decrease in Eos in peripheral blood was accompanied by an improvement in FEV1 and quality of life (ACQ-5), especially in the group of patients with nasal polyposis. Reslizumab also have shown a decrease in exacerbations, an improvement in quality of life, and a FEV1 improvement [31,32,33].

Real-life studies with reslizumab confirm the data obtained from clinical trials in the treatment of severe eosinophilic asthma. Observational post-marketing studies have shown a consistent reduction in the frequency of exacerbations, improvements in lung function and quality of life, and a reduced need for oral corticosteroids [32].

Moreover, there are data suggesting that reslizumab could be useful in critical cases of asthma in the ICU [34]. The safety profile observed in real-world settings is consistent with clinical trial data. Common adverse effects include headaches, infusion-related reactions, and potential hypersensitivity reactions [29,35].

##### Anti-IL-5R Biologics

*Benralizumab*

Humanized monoclonal antibody that binds to the alpha subunit of the IL-5 receptor, preventing its activation and inducing the direct elimination of eosinophils and basophils through NK cells. It is administered subcutaneously at a dose of 30 mg of benralizumab every 4 weeks for the first 3 doses and then every 8 weeks [36,37].

The evaluation of the efficacy of benralizumab comes mainly from 2 studies of 48 and 56 weeks duration (CALIMA [38] and SIROCCO [39]), In the SIROCCO and CALIMA studies, benralizumab has demonstrated statistically significant reductions in the rate of exacerbations of 51% and 28%, respectively compared to placebo in the subpopulation with baseline plasma eosinophil levels ≥300/µL.

Its efficacy has been demonstrated in eosinophilic SA in terms of reducing severe exacerbations compared to placebo by up to 55%, improving symptom control, and reducing and discontinuing treatment with OCS, as well as increasing lung function (FEV1) from the baseline visit by 25% (398 mL) [37,38,39,40]. The ZONDA study [39], the dose of OCS were reduced for severe corticosteroid-dependent asthmatics, maintaining control in the majority (75% dose reduction) and even interrupting its use (in 52% of patients). The greatest benefits observed in patients with counts >300 Eos/l in peripheral blood and >150 Eos/l if they were patients with maintenance OCS.

Benralizumab showed greater efficacy in patients included the using OCS, concomitat nasal polyposis, low lung function, frequent exacerbations, and those with a long term disease [41,42,43]. The PONENTE study [44] showed a withdrawal of OCS in 62.5% of patients. The reduction occurred regardless of the baseline Eos value and a reduction in exacerbations was achieved [43].

It has proven to be a safe drug, which does not cause significant or frequent adverse reactions. The most frequently reported adverse reactions during treatment are headache (8%) and pharyngitis (3%). Treatment with anti IL-5 and anti IL-5Rα antibodies may be associated with the development of neutralizing antibodies. According to the available data, it does not present drug interactions [36].

Real-world studies reinforce results obtained premarketing studies. Benralizumab is an effective and safe therapeutic option for patients with severe eosinophilic asthma who do not achieve adequate control with conventional treatments. Many real-world studies report similar outcomes to those seen in clinical trials supporting its ability to reduce exacerbations, improve lung function, decrease dependence on oral corticosteroids, and enhance patients’ quality of life. Overall, benralizumab stands out as a valuable intervention in real-world clinical practice, offering significant clinical benefits and a favorable safety profile. Adverse effects of benralizumab in real-world settings aligns with clinical trial data, with most adverse reactions being mild to moderate [45,46,47,48].

#### 1.2.3. Anti-IL-4 and -IL-13 Biologics

##### *Dupilumab* 

This recombinant human IgG4 monoclonal antibody inhibits IL-4 and IL-13 signaling through the Type I receptor (IL-4Rα/γc), as well as IL-4 and IL13 signaling through the Type II receptor (IL-4Rα/IL-13Rα). It is known that IL-4/IL-13 play a role in the development of eosinophilic airway inflammation by increasing VCAM-1 expression on vascular endothelial cells; the expression of CCR3 ligands, contributing to eosinophil migration into the airways; and the expression of periostin, which can activate eosinophils in the airways. IL-13/IL-4 inhibition by dupilumab leads to bronchial epithelial cell differentiation and mucus production reduction, also reducing airway smooth muscle cell contractility.

The recommended dose of dupilumab for adults and adolescents (12 years and older) is as follows: for patients with severe asthma taking oral corticosteroids or for patients with severe asthma and moderate to severe comorbid atopic dermatitis or adults with severe comorbid chronic rhinosinusitis with nasal polyposis, an initial dose of 600 mg (two 300 mg injections), followed by 300 mg every two weeks administered by subcutaneous injection; for all other patients, an initial dose of 400 mg (two 200 mg injections), followed by 200 mg every two weeks administered by subcutaneous injection [49].

The most frequent adverse effects are reactions at the injection site (includes erythema, edema, pruritus, pain, and swelling), conjunctivitis, arthralgia, oral herpes, and eosinophilia. Dupilumab does not present important interactions with other medications [49].

The dupilumab development program for asthma included three pivotal randomized, double-blind, placebo-controlled, parallel-group, multicenter studies (DRI12544, QUEST, and VENTURE) with a treatment duration of 24 to 52 weeks and in which a total of 2888 patients aged ≥ 12 years were included) [49].

Wenzel et al. [50,51] demonstrated dupilumab’s effectiveness in patients with moderate to severe asthma with > 300 Eos/l in blood or more than 3% in sputum. Subsequently, in other study [50] on 769 patients for 24 weeks of treatment with doses of 200 and 300 mg and 16 weeks of follow-up they observed a decrease in exacerbations and an improvement in FEV1 at week 12 that was maintained until week 24, regardless of the baseline Eos value (categorized ≥ 300 or <300 cells/l).

Castro et al. [52] compared two doses of dupilumab (200 and 300 mg) and placebo every two weeks throughout the 52 weeks of the study. The authors concluded that the treatment significantly reduced exacerbations compared to placebo, and improved lung function and asthma control. The benefits were greater in patients with eosinophilia ≥300 cells/l. The benefits were more evident in those patients with high T2 markers (≥150 Eos/mm^3^ and baseline FENO ≥ 25 ppb).

Two studies [53] showed that dupilumab reduced rates of severe exacerbations, improved FEV1 and asthma control, as well as suppressed type 2 inflammatory biomarkers in patients with SA, and independently of the presence or not of chronic rhinosinusitis (with or without nasal polyposis). In other research [54] dupilumab proved to decreased OCS consumption, and severe exacerbations and improvement in FEV1 in OCS-dependent patients. Baseline Eos count and the FENO were the best response predictors to the drug.

In the QUESTstudy [55], eosinophilia was recorded in 4.1% of the treated group versus 0.6% in the placebo group. The TRAVERSE study [55] a long-term safety and efficacy of the drug was proved. Finally, dupilumab may be effective in other comorbidities associated with SA such as atopic dermatitis and/or polyposis [56,57,58,59].

Real-life studies showed that dupilumab is an effective and safe treatment option for patients with severe asthma, particularly those with a type 2 inflammation profile. As in clinical trials, the evidence supports its role in reducing exacerbations, improving lung function, decreasing reliance on OCS, and enhancing overall quality of life. Dupilumab’s favorable safety profile and consistent efficacy in real-world settings make it a valuable addition to the therapeutic arsenal for severe asthma management [60,61]. Overall, dupilumab provides significant clinical benefits in a broad patient population, reinforcing its use as a cornerstone therapy for severe asthma in clinical practice. Real-world studies have included diverse patient populations, highlighting efficacy across various demographics and asthma phenotypes, including those with elevated eosinophil counts and those with non-eosinophilic asthma. There is a noted decrease in healthcare resource utilization, including fewer emergency department visits and hospitalizations related to asthma exacerbations after starting dupilumab [61,62,63,64].

These studies collectively underscore the benefits of dupilumab in managing severe asthma in real-world settings, affirming its role as an effective treatment option for eligible patients.

#### 1.2.4. Anti-TSLP Biologics

##### *Tezepelumab* 

This monoclonal antibody was approved as an additional maintenance treatment of SA that is not adequately controlled despite the administration of high-dose inhaled corticosteroids in combination with another medication for maintenance treatment [65,66]. The recommended dosage is 210 mg administered subcutaneously every 4 weeks [67]. Tezepelumab is a monoclonal antibody directed against TSLP, preventing the interaction with its heterodimeric receptor. In asthma, both allergic and nonallergic triggers induce TSLP production. The blockade of TSLP with tezepelumab reduces a broad spectrum of biomarkers and cytokines associated with airway inflammation (e.g., blood eosinophils, airway submucosal eosinophils, IgE, FeNO, IL-5, and IL- 13) [65].

The evaluation of the efficacy of tezepelumab is based on three studies: two 52-week studies whose primary objective was to evaluate the effect on reducing the annual rate of SA exacerbations (NAVIGATOR and PATHWAY) and a supporting study of 48 weeks designed to evaluate the effect of tezepelumab on reducing OCS (SOURCE). In the overall population, PATHWAY and NAVIGATOR patients receiving tezepelumab had significant reductions in the annual rate of severe asthma exacerbations compared with the placebo. In PATHWAY and NAVIGATOR, severe asthma exacerbations requiring emergency room visits and/or hospitalization were reduced by 85% and 79% with 210 mg Tezepelumab via subcutaneous C4W, respectively [66].

In absolute terms, in the NAVIGATOR study, a decrease of 1.17 exacerbations per patient per year was observed (reduction of 117 exacerbations per 100 patients in one year), while in the PATHWAY study, a decrease of 0.52 exacerbations was observed per patient per year (reduction of 52 exacerbations per 100 patients in one year).

In NAVIGATOR, tezepelumab demonstrated a reduction in severe asthma exacerbations (SAEs) independent of baseline blood eosinophil levels, FeNO, as well as allergic status. Similar results were observed in PATHWAY. In NAVIGATOR, the reduction in SAEs was greater with higher baseline blood eosinophil counts and FeNO values. The most common adverse effects were nasopharyngitis, upper respiratory tract infection, headache, and worsening asthma. All of them occurred with the same frequency in both treatment groups, except for asthma, which had a lower incidence in the tezepelumab group (7.4%) compared to the placebo group (15.7%). Overall, similar results were observed in the SOURCE study [67,68,69].

Tezepelumab would represent a treatment option compared to omalizumab in T2 allergic asthma, and a treatment option compared to benralizumab, mepolizumab, dupilumab and reslizumab in T2 allergic and/or eosinophilic asthma.

Tezepelumab is also a preferred treatment option in non-T2 asthma (non-allergic, non-eosinophilic asthma) with normal or low levels of eosinophils (<150 cells/μL) and FeNO (<25 ppb).

Real-world studies support that tezepelumab is a highly effective and safe treatment option for patients with severe asthma, including those with both type 2 and non-type 2 inflammatory profiles. Its broad mechanism of action, targeting upstream of the inflammatory cascade, allows it to reduce exacerbations, improve lung function, and decrease the need for oral corticosteroids across a wide spectrum of asthma patients. Tezepelumab’s consistent efficacy and favorable safety profile make it a valuable addition to the treatment landscape for severe asthma, particularly for those who have not responded adequately to other biologic therapies [70,71,72,73,74]. These references provide a comprehensive overview of the real-world data surrounding tezepelumab, highlighting its clinical benefits and safety profile in severe asthma management.

## 2. Discussion

There are several biological drugs currently available to treat severe asthma: anti-IL-5 (mepolizumab, reslizumab, benralizumab), anti-IgE (omalizumab), anti-IL-4/IL-13 (dupilumab), and anti-TSLP (tezepelumab) agents (Table 1).

**Figure 1:**
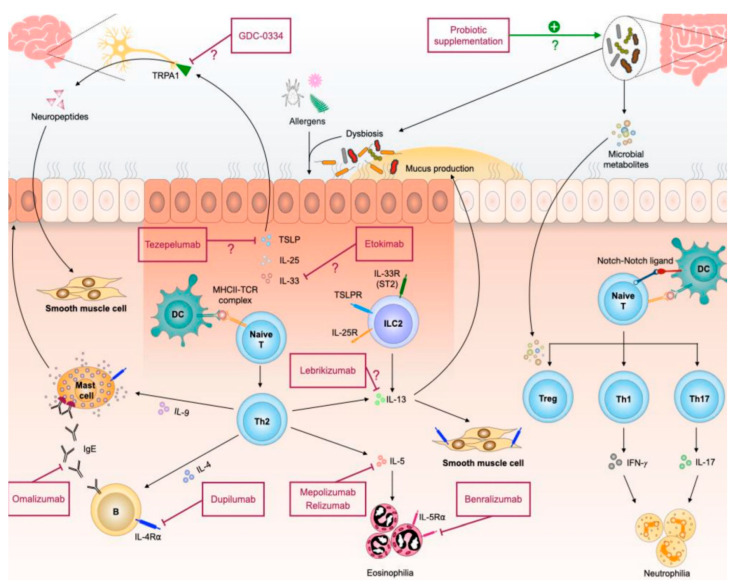
Pathogenesis of allergic inflammation and therapeutic interventions that are currently available and those with potential (Taken from Chiu CJ et al. *Int. J. Mol. Sci.* 2021; 22:4528) [73].

All of them are usually prescribed by multidisciplinary teams for patients who frequently suffer from exacerbations and show signs of T2 inflammation, although tezepelumab is also recommended for the treatment of those with non-T2 inflammation (Figure 1).

As described above, currently, we have sufficient real-life data to corroborate the good results obtained in clinical trials by these type of drugs for severe asthma patients. Biologics have significantly changed the management of severe asthma, particularly for patients with eosinophilic asthma or those with high IgE levels.

Overall, observational studies reveal their good results in terms of efficacy, reducing exacerbations and leading to fewer emergency room visits and hospitalizations. Also, they improve quality of life with better asthma control and overall quality of life, with reductions in symptoms and better functional status. Moreover, they have proven to be safe drugs. Generally well tolerated, they exhibit mild to moderate adverse events, with the most common being injection site reactions and headache. In the long term, data suggest a favorable safety profile, though ongoing monitoring is essential for these patients.

Deciding which biologic is most suitable for each patient is sometimes not easy. Multidisciplinary teams, following the protocols proposed by the clinical practice guidelines for severe asthma (GEMA [2] and GINA [3]), establish the individualized treatment for each patient. Likewise, if necessary, they decide when to change it and what new biological therapy the patient should receive. Firstly, inflammatory biomarkers (blood eosinophils and FeNO) and the allergic disease must be taken into account. However, patient preference, the route of administration, the frequency of dosing, and cost must also be taken into consideration [74,75].

The complex management of patients with severe asthma necessitates follow-up in specialized multidisciplinary units. The essential members of the team will be respiratory/allergy and hospital pharmacist specialists as well as asthma specialist nurses with appropriate training and experience in SA. On this basis, other specialists are required to work in a coordinated manner to rule out and adequately treat all comorbidities or aggravating factors of the patient’s asthma: otorhinolaryngologists, respiratory physiotherapy specialists, pediatricians (transition from children with SA to adult units), psychologists–psychiatrists, physiotherapists–rehabilitators, digestive specialists, dietitians, endocrinologists, and community pharmacists. All of them are important for the better quality of care that can be offered to SA patients.

## 3. Conclusions

The emergence of new biological treatments for patients with severe asthma has meant a paradigm shift and an improvement in the personalized management of the disease.

Selecting the most suitable biologic for each patient efficiently is a challenge that clinicians should address in order to optimize these treatments in a personalized, efficient way. Further studies are needed to address different aspects that have not yet been clarified. New predictors, T2 and non-T2 inflammation response markers, studies on treatment duration, and discontinuation criteria are needed.

### Future Directions

Future treatments for severe asthma will focus on new therapeutic targets or cytokines. There are many new biologics under investigation, targeting different interleukins: anti-Il-33 biologics such as itepekimab and tozorakimab, anti-IL-23 biologics, a cytokine that promotes Th17 cell proliferation, neutrophil recruitment, and T2 cytokine production such as risankizumab.

Another new therapeutic target under study is TL1A, a member of the TNF ligand superfamily. It binds to the DR3 receptor, which is constitutively expressed at low levels on the surfaces of T, B, and NK cells. This binding is important for sustained pathological T2 responses. Blocking it with neutralizing antibodies reduces inflammation and the levels of IL-4, IL-5, and IL-13 cytokines. A new biologic of this type, TL1A, allows for the very potent and selective neutralization of DR3 signaling and is potentially useful for the treatment of diseases involving TL1A deregulation, including diseases with a fibrotic component, such as asthma. These drugs could be useful in severe non-T2 asthma, for whose patients there are not many therapies available.

Another future strategy will be the design of new forms of administration that would improve therapeutic concordance, efficacy, and side effects by reducing systemic impact. One example of this new administration route is ecleralimab, an inhaled biologic anti-TSLP that is currently under investigation.

On the other hand, SA multidisciplinary teams, in which patients themselves participate and engage, can play a crucial role in the management of this complex condition. The involvement of patients is key, as their first-hand experience with the condition can offer valuable insights leading to improved treatment outcomes. Expert patient platforms, particularly in SA, are especially useful in this context. These platforms allow patients to share knowledge, exchange experiences, and provide peer support, fostering a more patient-centered approach to care.

## Figures and Tables

**Table 1:** Biologics for severe asthma treatment. (Adapted from: https://www.ema.europa.eu/en/documents/product-information. Accessed on 20 October 2024).

Name	Target	Biological Effects	Dosing	Common Adverse Effects	Therapeutic Indications
Omalizumab (Xolair^®^)	Fcε RI binding site of IgE	Decrease circulating total IgE. Decreased expression of FcεRI on inflammatory cells. Decreased mediator release.	According to IgE levels Every 2–4 wks (s.c.)	Pharyngitis, headache, anaphylactic reaction, abdominal pain, arthralgia, and injection site reactions.	Therapeutic indications: Allergic asthma (6 to <12 years of age). Chronic rhinosinusitis with nasal polyps (over 18 years of age). Chronic spontaneous urticaria (12 years of age and older).
Mepolizumab (Nucala^®^)	IL-5	Inhibits the bioactivity of IL-5 by blocking its binding to IL-5Rα complex expressed on the eosinophil cell surface. Reduces the production and survival of Eosinophils.	100 mg Every 4 wks (s.c.)	Respiratory and urinary infections, pharyngitis, herpes zoster, hypersensitivity reactions, headache, nasal congestion, abdominal pain, eczema, arthralgia, and administration-related reactions.	Severe eosinophilic asthma (>6 years). Chronic rhinosinusitis with nasal polyps (>18 years). Eosinophilic granulomatosis with polyangiitis (EGPA) (>6 years). Hypereosinophilic syndrome (>18 years).
Reslizumab (Cinqair^®^)	IL-5	Inhibiting IL-5 signaling. Decreased eosinophils in blood and sputum.	3 mg/kg Every 4 wks (i.v.)	Blood creatine phosphokinase increase.	Severe eosinophilic asthma (>18 years).
Benralizumab (Fasenta^®^)	IL-5R_	Decreased eosinophils and basophils.	30 mg Every 8 wks (s.c.)	Pharyngitis, hypersensitivity reactions, headache, and injection site reaction.	Severe eosinophilic asthma (>18 years).
Dupilumab (Dupixent^®^)	IL-4R_	Blockade IL-4/IL-4Rα binding. Blockade IL-13/IL-4Rα binding.	300 mg Every 2 wks (s.c.)	Conjunctivitis, oral herpes, arthralgia, and injection site reactions.	Atopic dermatitis (>6 months). Asthma (>6 years). Chronic rhinosinusitis with nasal poliposis (>18 years). Prurigo nodularis (>18 years). Eosinophilic esophagitis (>12 years). COPD (>18 years).
Tezepelumab (Tezspire^®^)	TSLP	TSLP blockade.	210 mg/kg Every 4 wks (s.c.)	Pharyngitis, rash, arthralgia, and injection site reaction.	Severe asthma (>12 years).

## Data Availability

No new data were created or analyzed in this study.
